# Dual captures of Colorado rodents: implications for transmission of hantaviruses.

**DOI:** 10.3201/eid0604.000406

**Published:** 2000

**Authors:** C H Calisher, J E Childs, W P Sweeney, K M Canestrop, B J Beaty

**Affiliations:** Arthropod-Borne and Infectious Diseases Laboratory, Clorado State University, Fort Collins, USA. calisher@promedmail.org

## Abstract

We analyzed dual-capture data collected during longitudinal studies monitoring transmission and persistence of Sin Nombre virus in rodents in Colorado. Our data indicate that multiple captures (two or more rodents captured in a single trap) may not be random, as indicated by previous studies, but rather the result of underlying, species-specific social behavior or cohesiveness. In the pairs we captured, most often, rodents were of the same species, were male, and could be recaptured as pairs. Therefore, dual captures of rodents, which are unusual but not rare, tend to occur among certain species, and appear to be nonrandom, group-foraging encounters. These demographic and ecologic characteristics may have implications for the study of the transmission of hantaviruses.


### Dual Captures of Colorado Rodents: Implications for Transmission of Hantaviruses

**Charles H. Calisher,* James E. Childs,† William P. Sweeney,* K. Max Canestorp,‡ and Barry J. Beaty***
*Colorado State University, Fort Collins, Colorado, USA; ‡Centers for Disease Control and Prevention, Atlanta, Georgia, USA; ‡Colorado Fish and Wildlife Assistance Office, Denver, Colorado, USA

We analyzed dual-capture data collected during longitudinal studies monitoring transmission and persistence of Sin Nombre virus in rodents in Colorado. Our data indicate that multiple captures (two or more rodents captured in a single trap) may not be random, as indicated in previous studies, but rather the result of underlying, species-specific social behavior or cohesiveness. In the pairs we captured, most often, rodents were of the same species, were male, and could be recaptured as pairs. Therefore, dual captures of rodents, which are unusual but not rare, tend to occur among certain species, and appear to be nonrandom, group-foraging encounters. These demographic and ecologic characteristics may have implications for the study of the transmission of hantaviruses.

The deer mouse, *Peromyscus maniculatus*, has been identified as the primary rodent reservoir of a newly identified virus responsible for the 1993 outbreak of severe respiratory disease in the southwestern United States (1). This virus, named Sin Nombre virus (SNV; family Bunyaviridae, genus *Hantavirus*) presumably is transmitted between rodents and to humans by inhalation of virus-contaminated aerosols of urine, saliva, and fecal material shed by subclinically infected rodents (2). Biting may also play an important role in rodent-to-rodent transmission (3).

We established longitudinal studies at three Colorado sites in 1994 to monitor SNV transmission and persistence in rodent populations and to assess factors that might influence virus transmission. On multiple occasions, two rodents were captured in a single trap. We summarize and analyze multiple-capture data and compare them with such data reported earlier (4-9). Our data indicate that rather than occurring as random encounters, dual captures may suggest underlying social behavior or cohesiveness that varies with species.

Address for correspondence: Charles H. Calisher, Arthropod-Borne and Infectious Diseases Laboratory, Foothills Campus, Colorado State University, Fort Collins, Colorado, USA; fax: 970-491-8323; e-mail: calisher@promedmail.org.

### Methods

### Description of Sites

Study areas in western Colorado, at Fort Lewis (La Plata County, southwestern Colorado; N 37° E 13' 30.9" latitude, W 108° E 10' 51.1" longitude, elevation 2,438 m) and Molina (Mesa County, west-central Colorado; N 39°E 09' 45.8" latitude, W 108°E 03' 18.4" longitude, elevation 1,951 m) were chosen because they were near residences of case-patients during the 1993 hantavirus outbreak. We established trapping webs at both sites (10). At Piñon Canyon Maneuver Site in southeastern Colorado, four sites were trapped. Trapping webs were used in a pinyon-juniper–short grass prairie habitat (N 37° E 33.024', W 103° E 59.560', elevation 1,585 m) and at the head of a canyon (N 37° E 32.754', W 103° E 49.343', elevation 1,524 m); trapping grids were used within that canyon (N 37° E 32.193', W 103° E 49.125', elevation 1,341 m) and at a functioning windmill (N 37° E 31.327', W 103° E 53.545', elevation 1,585 m).

At Fort Lewis, the habitat is montane shrubland (11) superimposed on intrusive igneous rocks forming laccoliths (12). Vegetation is predominantly composed of ponderosa pine (*Pinus ponderosa*), Gambel's oak (*Quercus gambeli*), a variety of grama grasses (*Bouteloua* spp.), and many other, more minor, floral components. At the Molina site, the habitat is semidesert shrubland (11) superimposed on Mancos shale (12). Vegetation includes juniper (*Juniperus* spp.), pinyon pine (*Pinus edulis*), and various shrubs and grasses. The Piñon Canyon site is managed by the Directorate of Environmental Compliance and Management, U.S. Department of the Army, Fort Carson, Colo. This site is a short grass prairie–pinyon-juniper community (13) with topographic features consisting of broad, moderately sloping uplands bordered by the Purgatoire River Canyon on the east, limestone hills on the west, and an extruded basalt hogback ridge on the south.

### Sampling Methods

Under license of the Division of Wildlife, Colorado Department of Natural Resources, we sampled for 3 days each 6 weeks, as weather permitted. Webs were established as follows: Twelve 7.6-cm x 8.9-cm x 22.9-cm noncollapsible traps (H.B. Sherman Traps, Inc., Tallahassee, Fla.) were placed on the ground at 5-m intervals for 20 m and then at 10-m intervals for 80 m, in each of 12 rows; an additional trap was placed at the central point, for a total of 145 traps in each web. Each trap's location was marked with a construction flag. Traps were baited with a mixture of cracked corn, oats, and peanut butter (6:2:1) and allowed to remain open overnight and then throughout the remainder of the trapping period. When temperatures were expected to be <5C, cosmetic balls of nonabsorbent material were placed in each trap, so that trapped animals would retain heat until they were processed. Each morning, traps were examined, and rodents were taken to a central processing area, where they were identified, weighed, and bled by inserting a capillary tube into the retroorbital plexus, before release at the capture site. We followed standard methods for sampling rodents and minimizing hazards from potentially infected animals (14). The handling and processing of rodents by these methods do not have a substantial impact on the subsequent survival or probability of recapturing most species (15-16). Each trap in which a rodent was captured was washed thoroughly with a mild solution of detergent to sterilize virus-contaminated material, then rinsed thoroughly and air-dried before being replaced in the field. This cleaning removed or diminished scent cues deposited by former inhabitants that could influence successive captures.

### Data Analysis

We first tested the null hypothesis that dual captures occurred at similar proportions across all sites. The observed number of dual captures per species was compared with the expected number of dual captures, obtained by multiplying the total capture events (single plus dual captures) for each species by the proportion of total trap events for all species that yielded dual captures (the total dual captures for all species [N=43] divided by the total capture events of all species [N=3972]). Four species (*Sigmodon hispidus* = hispid cotton rat, *Tamias minimus* = least chipmunk, *Chaetodipus hispidus* = hispid pocket mouse, and *Perognathus flavus* = silky pocket mouse) with fewer than five expected dual captures but one or more observed dual captures were not evaluated further. Twelve species with no dual captures and few total captures were excluded from most analyses:147 white-throated woodrats (*Neotoma albigula*), 142 white-footed mice (*P. leucopus*), 66 Ord's kangaroo rats (*Dipodomys ordii*), 65 northern grasshopper mice (*Onychomys leucogaster*), 38 northern rock mice (*P. nasutus*), 30 Mexican woodrats (*N. mexicana*), 9 southern plains woodrats (*N. micropus*), 9 brush mice (*P. boylii*), 8 house mice (*Mus musculus*), 3 Colorado chipmunks (*T. quadrivittatus*), and 3 meadow voles (*Microtus pennsylvanicus*) (Table 1). After testing (overall chi square), the observed proportion of dual capture events was compared with the expected value, after 95% Bonferroni confidence intervals (95% CI) for observed values were obtained (17). In these analyses, an upper standard normal table value corresponding to a probability tail of á/2$\underline{k}$ ($\underline{Z}_{á/2\underline{k}}$) was selected, where á = 0.05 and $\underline{k}$ = four comparison groups (deer mouse, *P. truei* = pinyon mouse, *Reithrodontomys megalotis* = western harvest mouse, and other). Where expected values fell outside the derived 95% CI, significant differences among dual captures of individual or grouped species were indicated. We followed the method of Taulman et al. (9) and Slade (18) to distinguish rodents in dual captures according to gender and age (adult

Table 1. Rodents involved in dual captures from three Colorado study sites (two western, one eastern)

| | Species[a] | | | | | | | |
|---|---|---|---|---|---|---|---|---|
| | P. man | P. truei | R. meg. | S. hisp. | T. min. | C. hisp. | Pe. flav. | Other[b] |
| Site (trap nights) | S (D) | S (D) | S (D) | S (D) | S (D) | S (D) | S (D) | S |
| Fort Lewis (10440) | 505 (5) | 6 (0) | 0 (0) | 0 (0) | 71 (1) | 0 (0) | 0 (0) | 2 |
| Molina (9135) | 566 (8) | 217 (0) | 2 (0) | 0 (0) | 132 (0) | 0 (0) | 0 (0) | 12 |
| Piñon Canyon (24375) | 829 (10[c]) | 561 (0) | 562 (15) | 259 (2) | 1 (0) | 23 (1) | 195 (1) | 520 |
| Total | 1,900 (23) | 784 (0) | 564 (15) | 259 (2) | 204 (1) | 23 (1) | 195 (1) | 534 |

[a]Numbers of single (S) and dual (D) captures are listed by species at each study site. P. man = *Peromyscus maniculatus*; P. truei = *Peromyscus truei*; R. meg. = *Reithrodontomys megalotis*; S. hisp. = *Sigmodon hispidus*; T. min. = *Tamias minimus*; C. hisp. = *Chaetodipus hispidus;* Pe. flav. = *Perognathus flavus.*
[b]534 rodents from 12 other species; no dual captures.
[c]One of these pairs was adult male deer mouse and an adult male pinyon mouse.

versus juvenile, based on weight). Animals were assigned to age classes according to body weight by using the values of Fitzgerald et al. (11).

### Results

### Dual Captures by Site

At Fort Lewis, 594 rodents belonging to two genera and three species were trapped in 10,440 trap nights between June 1994 and October 1997 (Table 1). Most single (86.4%) trap events and dual (5/6) captures were deer mice, but 71 least chipmunks, including one dual capture, were obtained. Two juvenile female deer mice were captured as a pair on successive days; one other female deer mouse captured as one of a pair had been captured alone the previous day (Table 2). One juvenile deer mouse was captured with an adult male deer mouse and then recaptured alone 5 months later. A dual capture of seropositive adult, male deer mice was made in October 1999 (data not included in these summaries). These two mice had been captured together in April 1999 and were seropositive at that time. Neither had been recaptured between April and October.

Table 2. Sex and age association for dually captured rodents

| | No. of species (recapture pairs)[b] | | | | | |
|---|---|---|---|---|---|---|
| Age–Sex[a] | P. man. | P. truei | R. meg. | S. hisp. | T. min | Ch. hisp. |
| AM/AM | 3 | 0 | 3 | 1 | 0 | 0 |
| AM/AF | 10 (1) | 0 | 5 (2)[c,d] | 0 | 0 | 1 |
| AM/JF | 2 | 0 | 2 | 0 | 0 | 0 |
| AF/JF | 1 | 0 | 1 | 0 | 0 | 0 |
| JM/JM | 2 | 0 | 2 | 0 | 0 | 0 |
| JF/JF | 3 | 0 | 1 (1) | 1 | 1 | 0 |
| JM/JF | 2 | 0 | 1 | 0 | 0 | 0 |

[a]A = adult (deer mouse 18 g; western harvest mice 9 g; hispid cotton rats 125 g; least chipmunks 30 g; hispid pocket mice 50 g; silky pocket mice 7 g. J = subadult or younger; M = male; F = female. Source: Fitzgerald et al., 1994 (11).
[b]For definition of species see Table l.
[c]Adult male captured with an adult female 03/11/97 and with a different adult female 04/20/97.
[d]Adult male capture with an adult female 04/19/97 and with a juvenile female 04/20/97.

At Molina, 945 rodents belonging to four genera and seven species were trapped in 9,135 trap nights between October 1994 and October 1997 (Table 1). Most single (61.3% of trap events) and all dual captures were deer mice. Two of the pairs of deer mice dually captured had been trapped individually the previous day; three of the other six deer mice dually captured also had been trapped alone previously (Table 2). No dual captures of pinyon mice were obtained, although 217 mice of this species (many of them later recaptured) were sampled.

At the Piñon Canyon site, 3,008 rodents belonging to 11 genera and 18 species were trapped in 24,375 trap nights between January 1994 and November 1997. Deer mice were the dominant rodents for single and dual captures (28.2%), although dual captures were also recorded for western harvest mice (15 dual captures), hispid cotton rats (2 dual captures), hispid pocket mice (1 dual capture), and silky pocket mice (1 dual capture). No dual captures of pinyon mice were obtained, although 561 mice were trapped individually. In addition, no dual captures were made among 534 mice of 12 other species trapped (Table 1). An adult male deer mouse and an adult female deer mouse were captured as a pair on sequential days (Table 2). Two pairs of deer mice, two pairs of western harvest mice, four western harvest mice, and five deer mice had been captured singly the previous day.

### Summary of Dual Captures

In all, 43 dual captures were made in 43,950 trap nights (Table 1), and the proportion of dual captures to all capture events (N=4,506) was 0.95% including all species and 1.08% excluding mice of the 12 species with no dual captures and too few total captures to be included (N=3,972). According to the overall proportion of dual captures to total capture events, the distribution of observed dual captures by species was different from that expected by chance, based on the overall proportion of dual captures to total capture events. Deer mice accounted for 42.8% of the 4,549 rodents captured, 42.7% of total capture events (48.4% when 12 species were removed from calculations), and 53.5% of the dual captures; western harvest mice represented 13.1% of the rodents, 12.8% of total capture events (14.6%), and 34.9% of the dual captures; pinyon mice represented 17.2% of all rodents captured and 17.4% of the capture events (19.7%), but no dual captures (one pinyon mouse was captured with a deer mouse); and mice of four other species included in further analyses represented 11.7% of all rodents captured and 11.9% of capture events (Tables 1 and 2; chi square = 21.67, df = 3, P<0.001). Comparisons of expected proportions of dual captures with Bonferroni 95% CIs of observed proportions indicated that pinyon mice were captured as pairs less frequently than expected by chance, while western harvest mice were captured as pairs more frequently than expected by chance (Table 3). Dual captures for deer mice and the category comprising four other species occurred at frequencies within the predicted range. The proportion of dual captures to total capture events by species (excluding the 12 removed) and across sites were consistent for deer mice (1.0%, 1.4%, and 1.2%) and pinyon mice (none at two sites).

### Species Considerations

Of 43 dual captures, 42 (97.7%) were conspecific; the sole exception was a dual capture of an adult male pinyon mouse and an adult male deer mouse. Using data from the Piñon Canyon study site, and excluding the 12 removed species, we calculated the probability of obtaining only single species dual captures, using the proportion that each species contributed to the total captures as the relative expected availability of that species for being dually captured. When this method and the binomial distribution were used, same species dual captures could be expected to comprise 0.282 of all dual captures (849 deer mice captured, 0.34 of total, probability of dual capture = 0.116; 561 pinyon mice captured, 0.26, 0.068; 592 western harvest mice, 0.24, 0.058; 486 hispid cotton rats, least chipmunks, hispid pocket mice, and silky pocket mice caught, 0.20, 0.04), yet 15 (93.8%) of 16 dual captures were of the same species (chi square = 34.3, df = 1, P<0.001).

### Sex Differences

Forty-five male rodents (52.3% of the dually trapped rodents) were involved in 34 (79.1%) of 43 dual captures; 41 female rodents (47.7%) were involved in 32 (74.4%) of 43 dual captures. Nineteen (44.2%) dual captures involved the same sex (11 pairs of male, 8 pairs of female rodents). Adult male and female deer mice and western harvest mice were caught more often as single-sex pairs than could be expected by chance (deer mice: chi square = 13.61, P<0.001; western harvest mice: chi square = 5.08, P = 0.02), although male and female deer mice (chi square = 0.00, P = 0.96) and western harvest mice (chi square 0.00, P = 0.95) were equally likely to be involved in dual captures.

Table 3. Numbers of dual captures among three rodent species

| | Observed | | Expected | | |
|---|---|---|---|---|---|
| Species | No. | % | No. | % | Bonferroni[a] 95% CI |
| *Peromyscus maniculatus* | 23 | 53.5 | 20.8 | 48.8 | 34.5%<P<72.5% |
| *P. truei* | 0 (1)[b] | 0.0 (2.3)[b] | 8.5 | 19.7 | 0.0%<P<8.0%[c] |
| *Reithrodontomys megalotis* | 15 | 34.9 | 6.3 | 14.6 | 15.8%<P<54.0%[d] |

[a]Differences occur between the observed and expected percentage of dual captures when the expected falls outside the 95% Bonferroni confidence interval (CI) of the observed proportion.
[b]One *P. truei* was captured with a *P. maniculatus*. Values in parentheses and those generated for expected and Bonferroni 95% confidence interval categories assume observed dual captures = 1.
[c]P<0.05 observed less than expected.
[d]P<0.05 observed greater than expected.

### Age Differences

A total of 36 (83.7%) dual captures involved animals of the same approximate age (23 pairs were adults, 13 pairs were juveniles, and 7 pairs were mixed; Table 2). Thirty-three juvenile rodents were involved in 20 (46.5%; 38.4% of the 86 rodents caught as pairs) of 43 dual captures but constituted only 17.5% of all captures (chi square = 25.8, df = 1, P<0.001). Adult deer mice were more likely than subadult deer mice (chi square 6.83, P = 0.009) to be involved in dual captures, but there was no significant difference between adult and subadult western harvest mice (chi square 1.61, P = 0.20). Adult females were never dually trapped with other adult females or with juvenile males, and adult males were not trapped with juvenile males. Of the 32 dual captures of rodents between October and April, the period in which 44.6% of the total trap nights and 49.1% of the rodents were captured, 12 (37.5%) involved juvenile rodents, compared with 8 (72.7%) of 11 rodents dually captured from May to September.

On three occasions, three deer mice were caught in a single trap. The first trio included two adult females and an adult male; one of the females and the male were captured together the next night and that female was captured almost 7 months later with a third female; none had antibody to SNV. An adult male, an adult female, and a partially cannibalized juvenile comprised the second trio; the male had antibody to SNV. An adult, seropositive female, an adult seropositive male, and a seronegative juvenile female comprised the third trio.

### Discussion

Our findings indicate that, at these sites and these times, dual captures of rodents were unusual but not rare and that some species were more or less likely to be captured as pairs. Also, pairs most often comprised rodents of the same species, and males were more often captured as pairs than females. Certain animals were captured as one of a pair on multiple occasions, and pairs of rodents were recaptured as pairs. In previous analyses of dual captures of rodents, Bergstrom (7) and Bergstrom and Sauer (8) suggested that multiple captures occurred as “random nonsynchronous encounters” of pairs of small mammals, rather than as social traveling. Further, these researchers suggested that such events occurred under the following conditions: interspecific multiple captures in one trap; a higher spring weight in traps that capture more than one animal compared with traps with single captures; random sex-age associations of animals captured together; no recaptures of pairs once caught together; adults captured with juveniles; and increased numbers of double captures in areas with higher population density. A design using side-by-side traps or one using entry timers might provide collateral information, but the closing of a door on a trap might also frighten away a nearby rodent.

Our results discount some of the general statements listed above. Only once did we capture a pair of rodents of different species, and the number of same-species dual captures at the Piñon Canyon site far exceeded the expected number if rodents of each species were available in proportion to their capture frequencies and behaved randomly. In addition, locally abundant species, such as pinyon mice, were never captured as same-species pairs. Therefore, our findings do not support a null hypothesis of random species mixing among dual captures in these areas.

Getz (4), studying multiply captured *Microtus pennsylvanicus* in a Wisconsin marsh, found no indication of significant antagonism between adult and immature males, at least during the declining phase of the population cycle. In addition, he was able to capture many adult female-immature male pairs. Although he recorded more than 750 instances of dual captures, and some animals were captured together as many as six times, he concluded that no formal social structure was indicated within this meadow vole population and that movement and association of individual voles within this population were random. Analyzing multiple capture data on several rodent species in a mixed desert-shrub and mesquite-grassland in northern Mexico, Petersen (5) found that most (90%) of the multiple captures were made during the breeding season, that most (75%) of the intraspecific double captures were heterosexual, and that of 12 dual captures of *R. megalotis*, all were male-female; no indication was given of the ages of the rodents. Because certain species at relatively high population densities were not captured dually, Petersen concluded that some sigmodontine rodents are more social than are heteromyid rodents. Blaustein and Rothstein (6) reported multiple captures of *R. megalotis* and concluded that the results of their studies (frequent capture as pairs, female-male combinations most prevalent, dual captures most common during the non-breeding season) suggested that this tendency may be adaptive with respect to predator avoidance and foraging.

Only once was the specific age-sex pairing of two adult females encountered in our study areas. Other researchers have also noted that this combination did not occur or occurred rarely (9). In contrast, adult male-adult female pairings occurred more often than expected by random assortment at our sites (Table 1) and those of Getz (4), Petersen (5), and Blaustein and Rothstein (6), suggesting that dual captures are not random among rodents of different sex and age classes in these communities. Different species communities sampled in other locations may show variant patterns. For example, although Fleharty and Mares's (19) data also indicated a single-sex predominance among dual captures of hispid cotton rats, these researchers obtained heterosexual pairs in only 14 of 50 double captures. Pairs of males were obtained on 26 occasions, pairs of females on 10, and a triple capture of males was obtained once. Obviously, specific age-sex pairing may vary by season, location, and species, and no general statement can be made about social traveling among rodents.

Odors of previous trap occupants can influence subsequent captures (11). Adult male and female deer mice may prefer traps baited with the odor of conspecifics during the breeding season, but outside the breeding season unscented traps are visited preferentially (20,21). Summerlin and Wolfe (22) suggested that dominant cotton rats are more susceptible to trapping than juveniles and that avoidance of traps visited by dominant rats may bias results toward adults. In our study, all traps in which rodents were captured were washed in dilute detergent before resetting and rinsed repeatedly and sequentially in buckets of fresh water to mitigate inhibition or attraction because of residual odors. Traps usually were not used for at least 3 weeks after they were washed. Such an interval likely would further serve to abate residual odors or disinfectant and mouse scents. In this regard, it has been shown that hypochlorite decontamination of traps does not influence trapping rates of rodents (23).

Although we did not test our trap spring weights, western harvest mice were captured as pairs more often than expected (Table 2). In Colorado, this species has an adult weight of >10 g, so that two adults weighed less than, or were similar in weight to, single adults of most other species caught at lower frequency. One would expect such an increase in the proportion of dual captures of smaller species if spring weight were a limiting factor. In addition, since western harvest mice (body length ca. 60 mm) are shorter than deer mice (body length ca. 90 mm), two western harvest mice entering in tandem would more likely be inside a trap when the treadle was tripped. This observation does not, however, rule out the possibility of social traveling but neither does it provide evidence to disprove such a hypothesis. These dually captured rodents might not have been traveling together, but group foraging certainly would put them in close proximity.

When our results were compared with those of Taulman et al. (9) (for their Sherman live traps only), the latter's ratio of double captures was 0.050/100 trap nights, with an overall trap success of 5.64 captures/100 traps. Our overall ratio of dual captures was nearly double, at 0.097/100 trap nights, but our trap success was substantially higher (8.94/100 traps). These proportions suggest that our higher ratio of dual captures was almost directly proportional to our higher overall trap results. Frequency of dual captures may increase with population density or trap success, as suggested by Bergstrom (7), Bergstrom and Sauer (8), and others. However, our findings indicate that dual captures do not occur randomly across species and demographic categories and support a hypothesis of nonrandom captures among the species and areas studied.

The influence of rodent behavior or spacing on hantavirus transmission has rarely been addressed (24). If dual capture trap success is an index of group traveling or cohesiveness, a reasonable first hypothesis is that species with higher degrees of social contact, or at least no aversion to it, have higher prevalences of infection with hantaviruses transmitted through close contact. Although data are fragmentary and alternative interpretations and caveats can be offered, in several studies on hantaviruses circulating in western rodent populations during nonepidemic periods, the prevalence of antibody to SNV was highest in western harvest mice (23%; infecting agent likely El Moro Canyon virus) and lowest in pinyon mice (3%), while deer mice maintained a middle position (11%) (25). Dual capture data from this study are intriguingly consistent with these data, suggesting that further observations on behavior and spacing among species are warranted. In our continuing studies, we will sample tissues of dually captured deer mice and determine their familial relationship using DNA microsatellites, a recently developed technique (W. C. Black IV and G. A. Kaufman, manuscript in preparation).

### Acknowledgments

We thank J.F. Taulman for valuable discussions; J. Jeffery Root for editorial advice; Ed Kuhn, Gordon Smith, Catherine Crabb, Robin Carns, Marcia Patterson, Heather Clifton, Ted Davis, and Edgar C. de Van III for invaluable field assistance; and the U.S. Army, Fort Carson, for allowing us to conduct studies at the Piñon Canyon Maneuver Site.

Funding for this work was provided by the Centers for Disease Control and Prevention, Atlanta, Ga., under cooperative agreement No. U50/ccu809862-03.

Dr. Calisher is professor of microbiology, Arthropod-Borne and Infectious Diseases Laboratory, Department of Microbiology, Colorado State University. His areas of expertise are arboviruses, hantaviruses, and other rodent-borne viruses. His research focuses on hantaviruses, arboviruses, arenaviruses, and epidemiology.

### References

1. Childs JE, Ksiazek TG, Spiropoulou CF, Krebs JW, Morzunov S, Maupin GO, et al. Serologic and genetic identification of *Peromyscus maniculatus* as the primary rodent reservoir for a new hantavirus in the southwestern United States. J Infect Dis 1994;169:1271-80.
2. Tsai TF. Hemorrhagic fever with renal syndrome: mode of transmission to humans. Lab Anim Sci 1987;37:428-30.
3. Glass GE, Childs JE, Korch GW, LeDuc JW. Association of intraspecific wounding with hantavirus infection in wild rats (*Rattus norvegicus*). Epidemiol Infect 1988;101:459-72.
4. Getz LL. Social structure and aggressive behavior in a population of *Microtus pennsylvanicus*. Journal of Mammalogy 1972;53:310-7.
5. Petersen MK. An analysis of multiple captures in several rodents from Durango, Mexico. Journal of Mammalogy 1975;56:703-5.
6. Blaustein AR, Rothstein SI. Multiple captures of *Reithrodontomys megalotis*: social bonding in a mouse? American Midland Naturalist 1978;100:376-83.
7. Bergstrom BJ. An analysis of multiple captures in *Peromyscus* sp. with a critique on methodology. Canadian Journal of Zoology 1986;64:1407-11.
8. Bergstrom BJ, Sauer JR. Social traveling inferred from multiple captures: testing assumptions. American Midland Naturalist 1986;115:201-3.
9. Taulman JF, Thill RE, Williamson JH. Double captures of small mammals in single-capture traps: random encounters or social traveling? Southwestern Naturalist 1994;39:358-63.
10. Anderson DR, Burnham KP, White GC, Otis DL. Density estimation of small-mammal populations using a trapping web and distance sampling methods. Ecology 1983;64:674-80.
11. Fitzgerald JP, Meaney CA, Armstrong DM. Mammals of Colorado. University Press of Colorado. Niwot, Colorado, 1994.
12. Baars DL. Navajo country: a geology and natural history of the Four Corners Region. Albuquerque, New Mexico: University of New Mexico Press; 1995. p.255.
13. Costello DF. Vegetation zones in Colorado. In: Harrington HD. Manual of the plants of Colorado. Chicago: Swallow Press, Inc; 1954. p. iii-x.
14. Mills JN, Childs JE, Ksiazek TG, Peters CJ, Velleca WM. Methods for trapping and sampling small mammals for virologic testing. Atlanta, Ga.: U.S. Dept. of Health and Human Services; 1995.
15. Swann DE, Kuenzi AJ, Morrison ML, DeStefano S. Effects of sampling blood on survival of small mammals. Journal of Mammalogy 1997;78:908-13.
16. Parmenter CA, Yates TL, Parmenter RR, Mills JN, Childs JE, Campbell ML, et al. Small mammal survival and trappability in mark-recapture monitoring programs for hantavirus. J Wildl Dis 1998;34:1-12.
17. Byers CR, Steinhorst RK, Krausman PR. Clarification of a technique for analysis of utilization-availability data. Journal of Wildlife Management 1984;48:1050-3.
18. Slade NA. Analysis of social structure from multiple capture data. Journal of Mammalogy 1976;57:790-5.
19. Fleharty ED, Mares MA. Habitat preference and spatial relations of *Sigmodon hispidus* on a remnant prairie in west-central Kansas. Southwestern Naturalist 1973;18:21-9.
20. Daly M, Wilson MI, Behrends P, Faux SF. Seasonally variable effects of conspecific odors upon capture of deer mice, *Peromyscus maniculatus gambelii*. Behaviorial Biology 1978;23:254-9.
21. Daly M, Wilson MI, Behrends P. Factors affecting rodent's responses to odours of strangers encountered in the field: experiments with odour baited traps. Behavioral Ecology and Sociobiology 1980;6:323-9.
22. Summerlin CT, Wolfe JL. Social influences on trap response of the cotton rat *Sigmodon hispidus*. Ecology 1973;54:1156-9.
23. Yunger JA, Randa LA. Trap decontamination using hypochlorite: effects on trappability of small mammals. Journal of Mammalogy 1999;80:1336-40.
24. Korch GW, Childs JE, Glass GE, Rossi CA, LeDuc JW. Serologic evidence of hantaviral infections within small mammal communities of Baltimore, Maryland: spatial and temporal patterns and host range. Am J Trop Med Hyg 1989;41:230-40.
25. Mills JN, Ksiazek TG, Ellis BE, Rollin PE, Nichol ST, Yates TL, et al. Patterns of association with host and habitat: antibody reactive with Sin Nombre virus in small mammals in the major biotic communities of the southwestern United States. Am J Trop Med Hyg 1997;56:273-84.
